# Carbon footprint and greenhouse gas emissions of different rice-based cropping systems using LCA

**DOI:** 10.1038/s41598-025-90157-2

**Published:** 2025-03-25

**Authors:** Mohammad Mofizur Rahman Jahangir, Eduardo Aguilera, Jannatul Ferdous, Farah Mahjabin, Abdullah Al Asif, Moutakin Hossan, Hassan Ahmad, Maximilian Bauer, Alberto Sanz Cobeña, Christoph Müller, Mohammad Zaman

**Affiliations:** 1 Department of Soil Science, Bangladesh Agricultural University, Mymensingh, 2202 Bangladesh; 2 Institute of Economy, Geography and Demography, Spanish National Research Council, 28006 Madrid, Spain; 3 Soil and Water Management & Crop Nutrition, Joint FAO/IAEA Division of Nuclear Techniques in Food & Agriculture, Vienna, Austria; 4 Department of Chemistry, Leibniz Universität Hannover, Hanover, Germany; 5 Institute of Plant Ecology (IFZ), Justus-Liebig University Giessen, Giessen, Germany; 6 Liebig Centre for Agroecology and Climate Impact Research, Justus Liebig University, Giessen, Germany; 7 School of Biology and Environmental Science and Earth Institute, University College Dublin, Belfield, Dublin 4, Ireland

**Keywords:** Carbon footprint, Co-designed Carbon footprint calculation tools, Greenhouse Gas (GHG) emissions, Major cropping patterns, Biochemistry, Biological techniques, Biogeochemistry, Climate sciences

## Abstract

There are many cropping systems on floodplain soils, but greenhouse gas (GHG) emission balances of these agricultural systems are rarely reported. Carbon (C) footprints of agricultural products were assessed using a co-designed life cycle assessment tool in major cropping systems in Bangladesh: rice-rice-rice (R-R-R/boro-aus-aman), rice-fallow-rice (R-F-R/boro-fallow-aman), maize-fallow-rice (M-F-R), wheat-mungbean-rice (W-Mu-R), and potato-rice-fallow (P-R-F) along with the field measurement of some of the systems. The rice system with dryland crops had higher nitrous oxide (N_2_O) emissions (3.8 in maize, 4.5 in potato and 0.92 kg N_2_O–N ha^−1^ in mungbean) than sole rice (0.73 in boro, 0.57 in aus and 1.94 kg N_2_O–N ha^−1^ in aman) systems but methane (CH_4_) emissions exhibited the opposite. Methane dominated, accounting for 50–80% of total emissions in rice systems. The boro rice-based systems (R-R-R and R-F-R) had the highest C footprint (ca. 25.8 and 19.2 Mg CO_2_e ha^−1^) while the P-F-R (12.3 Mg CO_2_e ha^−1^) and M-F-R (12.6 Mg CO_2_e ha^−1^) had the lowest C footprint. Boro and aus were more suitable to reduce C footprint. Measured CH_4_ and N_2_O data agreed well with the IPCC Tier 1 estimates but further study on GHG measurements in other agroecosystems and cropping systems are required to validate the estimation for adopting suitable GHG mitigation strategies.

## Introduction

Agriculture acts as the primary source of economic and food security for developing countries like Bangladesh. Increasing population and consumption are placing unprecedented demands on agriculture and natural resources in the region. We are confronted with one of the most difficult tasks of the twenty-first century: satisfying society’s expanding food demands while decreasing agriculture’s environmental impact^1^. With the advancement of the ‘Green Revolution’, the intensive use of different inputs such as synthetic fertilizers, herbicides, and insecticides have been established as a key strategy aiming for optimal productivity. The agricultural soils of Bangladesh have a deficit in all the nutrients since 1983–1984^2^ and latest identified limiting nutrient is manganese (Mn) in 2010. As a result, synthetic fertilizers, commonly urea, are used as the main source of nitrogen (N) to maintain crop growth, development, and higher yield. However, excessive N fertilizer supply can increase groundwater pollution, soil acidification and particularly the emissions of nitrous oxide (N_2_O), a potent greenhouse gas^3^ and ammonia (NH_3_), a major air pollutant^4^.

Rice, maize, and wheat are the major crops of Bangladesh, where rice is in the top position. In 2020, Bangladesh ranked third in the world in rice cultivation area and production^5^. Rice is a semi-aquatic plant, usually cultivated under complete flooded conditions, providing an anaerobic environment for methanogens and denitrifiers to degrade organic substances and reduce nitrate (NO_3_^−^), respectively^6^, which enhances greenhouse gas (GHG) emissions. Nitrogen loss with water is another key channel responsible for fertilizer N loss in paddy fields, including surface runoff, leaching, and lateral seepage, accounting for up to 50% of applied N fertilizer loss^7^. The N use efficiency (NUE) of rice, maize and potato is approximately 30–50%, 33%, and 40–50% respectively^8^ and the NUE in water spinach is 28% and 42% in white cabbage^9^. Organic inputs, such as crop residue, manures, and compost improve soil fertility, agricultural productivity, and crop yield by enhancing carbon (C) sequestration and nutrient mineralization in the soil^10,11^. However, organic amendments can cause GHG emissions through different processes such as priming, methanogenesis, nitrification, and denitrification^12^.

Rice-based production systems were reported to generate 523 million grams (Mg) of CO_2_e per year, accounting for 8.8–10.2% of total agricultural emissions globally in 2012^13^. In Bangladesh, GHG emissions from the agriculture sector increased by 80% in 1990–2017^14^. Furthermore, the country is a net importer of cereals, which is associated with imports of virtual land, water, and GHG emissions^15^. Agricultural emissions contributed to about 40% of the total emissions of Bangladesh in 2017–2019, using data from PRIMAP-cfr^16^ while CH_4_ from rice fields contributes about 7% of GHG emissions. According to FAOSTAT^17^, total soil N_2_O emissions between 1961 and 2020 grew from 1.5 to 4.2 kt N_2_O for manure application, from 4.4 to 11.2 kt N_2_O for crop residues and from 0.3 to 21.4 kt N_2_O for synthetic fertilizers. The estimation of agricultural emissions in the national GHG inventory of Bangladesh highly relies on IPCC (Intergovernmental Panel for Climate Change) Tier 1 methods, which are desk-based and cannot work as a standard for any specific region in which agriculture represents a significant share of total GHG emissions. Therefore, specific regional data on GHG emissions are necessary to understand and evaluate the contribution of agriculture to global warming.

The C footprint is a measure of the total amount of GHG emissions that is directly and indirectly caused by an activity or the life stages of a product, and it is generally calculated using Life Cycle Assessment (LCA), and is typically measured in terms of carbon dioxide equivalents (CO_2_e) taking the global warming potential (GWP) of each output into account. A potential answer to slow the pace of climate change could be found in the quantification and assessment of the degree of C emissions and energy consumption in an agroecosystem^18^. The C footprint is generally accepted and applied due to its importance in measuring environmental quality and management in agricultural sectors^19^ also in rice production^20^. Carbon sequestration or emissions depend on different factors, and they do not occur in a very specific or simultaneous manner. Due to seasonal variations in temperature and water regimes, varying lengths of crop growth, and variations in crop outputs (and yields), energy/feedstock use efficiencies, nutrient (fertilizer) inputs, residue/C returns, and other inputs influencing management activities and production, rice-based triple cropping systems have complex effects on GHG emissions^6^. An accounting of net GHG emissions along with C sequestration in soil is needed to evaluate strategies of GHG mitigation for rice-dominant cropping, which is a major contributor to the C footprint of global agriculture.

In Bangladesh, the LCA for C footprint has only been carried out for a specific rice-based cropping pattern^21^. Therefore, there is a lack of studies covering other types of rice-based cropping patterns in this country. In addition, there is a scarcity of measured and estimated data on GHG emissions from different cropping patterns, fertilization, and management practices. Thus, our main aim is to provide a first estimate of the C footprint of various rice-based cropping patterns in Bangladesh. The specific objectives are to (i) compare GHG emissions and the corresponding C footprint for individual crops in a season as well as for the whole pattern in a year, and (ii) to evaluate the crops, in particular, or the cropping system, as a whole, for sequestering C and mitigating C loss.

## Methods

The C footprint of the main products of major cropping systems in Bangladesh was assessed through an attributional LCA, using a co-designed calculation tool. The system boundaries were stablished “from cradle to farm gate”. The components of the GHG balance include upstream, direct, and downstream GHG emissions and the soil organic carbon (SOC) balance, expressed as CO_2_-equivalents (CO_2_e) using 100-year Global Warming Potential (GWP) factors from the IPCC’s 6th Assessment Report^22^. GHG emissions in the field were estimated using the IPCC Tier 1 method (global and regional) supplemented by field measurements of emissions from some treatments to assess the reliability of the estimated data. All components of the net primary production (NPP) in terms of dry matter, C and N were estimated to assess soil C and N inputs. Emissions were allocated between the main product and the residues based on their corresponding economic value. Description of the tool is provided in “Carbon footprint calculator” and “Soil organic carbon balance”.

### Carbon footprint calculator

All calculations of the LCA were made using a co-designed C footprint calculation tool. Its co-design was performed through an iterative process based on repeated feedbacks between developers and the participants of the FAO-IAEA’s Coordinated Research Project (CRP 20.150). In each meeting, developers explained the novel features and the users calculated C footprints from selected regions in their countries and suggested modifications to account for the specific features of their systems. The participants comprised of six research teams (1–4 persons in each team) from 6 countries including Vietnam, Bangladesh, Pakistan, Argentina, Costa Rica, and Ethiopia. The case studies covered field experiments and commercial farms with a wide variety of crop types and management practices, with an emphasis on rice paddies.

The tool is built in the Microsoft Excel environment to maximize the range of possible users and to allow for case-sensitive adjustments by the users. The tool has three main sheets: one for introducing crop data, one with emission factors and other coefficients (such as allometric and stoichiometric coefficients of the main crops) and one summarizing the results. Auxiliary sheets include soil data obtained from the Harmonized World Soil Database 2.0. climate data obtained from CRU TS 4^23^, and the electricity mix of each country, gathered from the World Development Indicators Database.

The basic crop data to be introduced includes information regarding location and main characteristics of the studied systems, intercropping period and management, crop and residue production, residue destiny shares (harvest, soil incorporation, burning, grazing), inputs of fertilizers, pesticides and electricity, number of passes of each machinery task, and prices of the products and residues. management, emissions, etc.). In the case of rice systems, water management information in the crop and intercrop period also has to be specified, as this affects the estimation of CH_4_ emissions and the soil organic C balance.

Crop coefficients include product and residue dry matter content, root:shoot ratio, product, residue and root C and N content over dry matter, and humification coefficients. Emission factors from the production of inputs (fertilizers, pesticides, machinery, fuel, electricity) are based on life cycle inventories (mainly Ecoinvent 3.0) and calculated with SimaPro software. Soil methane (CH_4_) and N_2_O emissions are estimated using Tier 1 methods from the revised 2006 Guidelines for National Inventories of the IPCC^24^, given that Tier 2 methods are not available for Bangladesh. In the case of N_2_O, this includes a disaggregation based on the type of fertilizer and the differentiation between rice and other crops cultivated in dry wet climates. In the case of CH_4_ emissions from rice fields, a default daily CH_4_ emission factor is modified by scaling factors considering the water regime during and prior to the cultivation period, the application of straw and external organic fertilizers, potential plant biomass growth is estimated with the NCEAS model, which is based on yearly water inputs (which in our case correspond to the sum of precipitation and irrigation). This potential biomass growth is scaled with qualitative information on weed management to estimate weed biomass production in the intercrop period and during the crop cycle. The calculation of the SOC balance is described in the next section. The tool is designed to ensure maximum flexibility regarding data availability while reporting the most reliable output. For example, if available, measured climate and soil data can be supplied to the tool but they can also be retrieved from global datasets. Crop residue, roots, and cover crop biomass are estimated with coefficients if no field measurements are available. In the same way, a prioritization procedure is implemented for the selection of GHG emission and C sequestration estimates, choosing measured data if they are available (i.e., Tier 2 data or Tier 1 estimates). However, in cases such as rice if measured data do not cover all studied treatments, measured data were only used to inform the selection of the best estimation method.

### Soil organic carbon balance

The SOC balance is calculated with the HSOC model^25^, a dynamic model built as a simplification of the RothC model^26^ with 2 active pools of SOC. This model has soil current SOC stocks, C inputs, input humification coefficients, and monthly temperature, soil water status, and soil cover as the main factors affecting the SOC balance. In this work, we modified the HSOC model in rice fields using the modifying factors for SOC mineralization rates to account for slower mineralization rates under flooded conditions. To facilitate the comparability of the data, we assumed that initial SOC content was equal to the SOC content in equilibrium in the most widespread of the studied rotations (Rice-Fallow-Rice), and all treatments were calculated as comparison to this value. In order to incorporate the SOC balance to the GHG balance and C footprint estimations, we ran the model for 100 years using the management and pedoclimatic data of each treatment and divided the result by 100 to get a yearly C sequestration rate. This rate was converted to CO_2_e using the molecular weight ratio of CO_2_ to C (3.67). This way, the reported SOC changes are in line with the other gases of the GHG emission balance, which are reported as 100-year GWP.

### Field data: cropping patterns and crop management

The experiments were carried out with five different rice-based cropping patterns where rice was cultivated as the main crop in annual double or triple rice systems or in rotation with other crops which is followed by most farmers under conventional cultivation practices. In Bangladesh, boro, aus and aman rice were grown in the mid-winter to pre-monsoon season (irrigated), monsoon season (no irrigation) and late monsoon season to winter (additional irrigation) (Table 1). Therefore, the cropping patterns were rice-rice-rice (R-R-R/boro-aus-aman), rice-fallow-rice (R-F-R/boro-fallow-aman), maize-fallow-rice (M-F-R), wheat-mungbean-rice (W-Mu-R), potato-rice-fallow (P-R-F). Each of the experiments were replicated thrice. Rice-fallow-rice (R-F-R) is the most widely used pattern in Bangladesh. Hence, R-F-R has been used as a reference in the estimation of SOC changes (described in Soil organic carbon balance section). The field sites have a noncalcareous dark grey floodplain soil (Aeric Haplaquept in the U.S. Soil Taxonomy) these soils are very deep and well drained and occur in agroecological zone 9 (AEZ-9; Old Brahmaputra Floodplain soil and the cropping patterns were R-F-R, R-R-R, and, W-Mu-R) AEZ-28 (Madhupur Tract, cropping pattern was M-F-R) and AEZ-4 (Karatoya Bangali Floodplain and the cropping pattern was P-R-F). The regions (AEZ-9, AEZ-28, AEZ-4) have a subtropical monsoon climate with a mean annual temperature of 26 °C, average annual rainfall of 1800–2200 mm, and relative humidity of 85% (Local weather stations). The observation data covered one annual cycle. The dominant regional soil type was Low Activity Clay (LAC) soil with 14–18% clay contents. There were also four dryland crops, wheat, maize, mungbean, and potato (Table 1). In boro, aus and aman seasons the age of seedlings was 42, 33 and 35 days, respectively. Wheat, maize, mungbean and potato were direct seeded crops. The rice fields experienced non-flooded preseason for less than 50 days before transplanting of seedlings in most of the rice-based cropping patterns. During the fallow period no crop was in the field but only spontaneous weed during ~ 12 weeks from May to August. Based on the Fertilizer Recommendation Guide, the rate of synthetic fertilizer application was determined for each of the crops. Urea, triple superphosphate (TSP), and Mourite of Potash (MoP), Gypsum was used as nutrient sources of N, P, K, S. No organic amendments were used besides crop residue and weed. In rice seasons the straw incorporation in soil ranged from 10 to 20% where it was 100% for potato. Regarding water management, aman was provided with supplemental irrigation whereas flood irrigation was used for boro and no irrigation for aus as there was enough rainwater during monsoon. Furrow irrigation was used for maize, and potato. Wheat requires about 1–2 irrigation events, but the land used in that experiment always remained in wet condition due to topography and shallow water table. Irrigation water was supplied from ground water by using electric pumps. Machinery was used for land preparation and spraying of solutions in the field for all the crop seasons. In each crop herbicides and pesticides were sprayed once in a season.

**Table 1:** Treatments of the experiments with their description.

Cropping patterns			Description		
Abbreviation	Elaboration	Growing duration	Growing area	Water management	Organic amendments
R-R-R	Rice (boro)—Rice (aus)—Rice (aman)	boro: Mid December to mid May aus: Mid May to mid August aman: August to December	AEZ-9 (Old Brahmaputra Floodplain, Aeric Haplaquept)	Supplementary irrigation for aman; flood irrigation for boro and no irrigation for aus	10–20% straw incorporation
R-F-R	Rice (boro)—Fallow—Rice (aman)	boro: Mid December to mid May aman: Late July to mid December	AEZ-9 (Old Brahmaputra Floodplain, Aeric Haplaquept)	Supplementary irrigation for aman; flood irrigation for boro	10–20% straw incorporation
M-F-R	Maize—Fallow—Rice (aman)	Maize: Mid December to May aman: Late July to December	AEZ-28 (Madhupur Tract)	Furrow irrigation for maize; flood irrigation for boro	30% crop residue incorporation
W-Mu-R	Wheat—Mungbean—Rice (aman)	Wheat: Mid November to Apirl Mungbean: April to July aman: August to November	AEZ-9 (Old Brahmaputra Floodplain, Aeric Haplaquept)	Light flood irrigation for wheat and mungbean; Supplementary irrigation for aman	15% crop residue for wheat and rice; 100% for mungbean
P-R-F	Potato—Rice (aman)—Fallow	Potato: Mid November to mid February aman: Late July to December	AEZ-4 (Karatoya Bangali Floodplain)	Furrow irrigation for Potato; Supplementary irrigation for aman	100% straw incorporation for potato; 30% for rice

### Greenhouse gas sampling

GHG measurements were conducted in boro rice, wheat and maize fields using the closed chamber technique^6,27^. The observation period began with the first application of urea under continuous flooded conditions and continued until emissions reached background levels. Chambers were made of soda glass and stainless-steel collars were placed on rice rows, covering four plants to a depth of 10 cm. Neoprene seals ensured an airtight connection between the chamber lid and the frame. Urea was applied inside the pre-installed collars using a broadcast method. Gas samples were collected at 0, 30, and 60 min after the camber set up during the day, between 10:00 a.m. and 4:00 p.m., on day 0, 1, 3, 5, 7, 10, 15, and 21 after each split urea application. A 60 mL Luer Lock syringe with a 25-gauge needle was used to collect 16 mL gas samples from the chamber headspace, which were then injected into pre-evacuated 12 mL vials. After storage for up to 7 days, the samples were analysed using a Varian 3800 gas chromatograph equipped with specific detectors for N_2_O, CO_2_, and CH_4_^6^.

### Soil and biomass sampling and analysis

Composite soil samples were taken from each replicated plot at a depth of 0–15 cm, using an auger, 4 days after the second split application of urea, which coincided with the peak of N_2_O emissions. The samples were collected from multiple locations near each GHG gas sampling chamber and stored in sealable plastic bags at 4 °C. In the field, soil pH was measured using a portable pH meter. A portion of the soil, after removing visible roots and litters through sieving with a 2 mm mesh, was analysed for ammonium (NH_4_^+^) and nitrate (NO_3_^-^) contents using the colorimetric method. Another portion of the soil was air-dried at room temperature (~ 25 °C) in the shade for two weeks and then processed (2 mm sieved) for analysis of SOC and total N (TN) using the wet oxidation method and Kjeldahl method, respectively (Jahangir et al., 2022). The values and corresponding sources used for the crops in this area are available in Begum et al.^28^ and Jahangir et al.^6^. To measure grain and residue biomass production, a 4 m^2^ area was chosen at random in the plot area just before harvest. The plants were cut at ground level, put in mesh bags, and left to air dry. The weights of the grain and crop residue were calculated after the grain was threshed from the sample. To assess the water content, a portion of the crop residue was oven dried at 65 °C for 72 h. Yields of crop residue were expressed on an oven-dry basis. Paddy grain yields were adjusted to 12% for rice and 14% moisture for wheat and maize.

## Results

### Net primary productivity

**Fig. 1:**
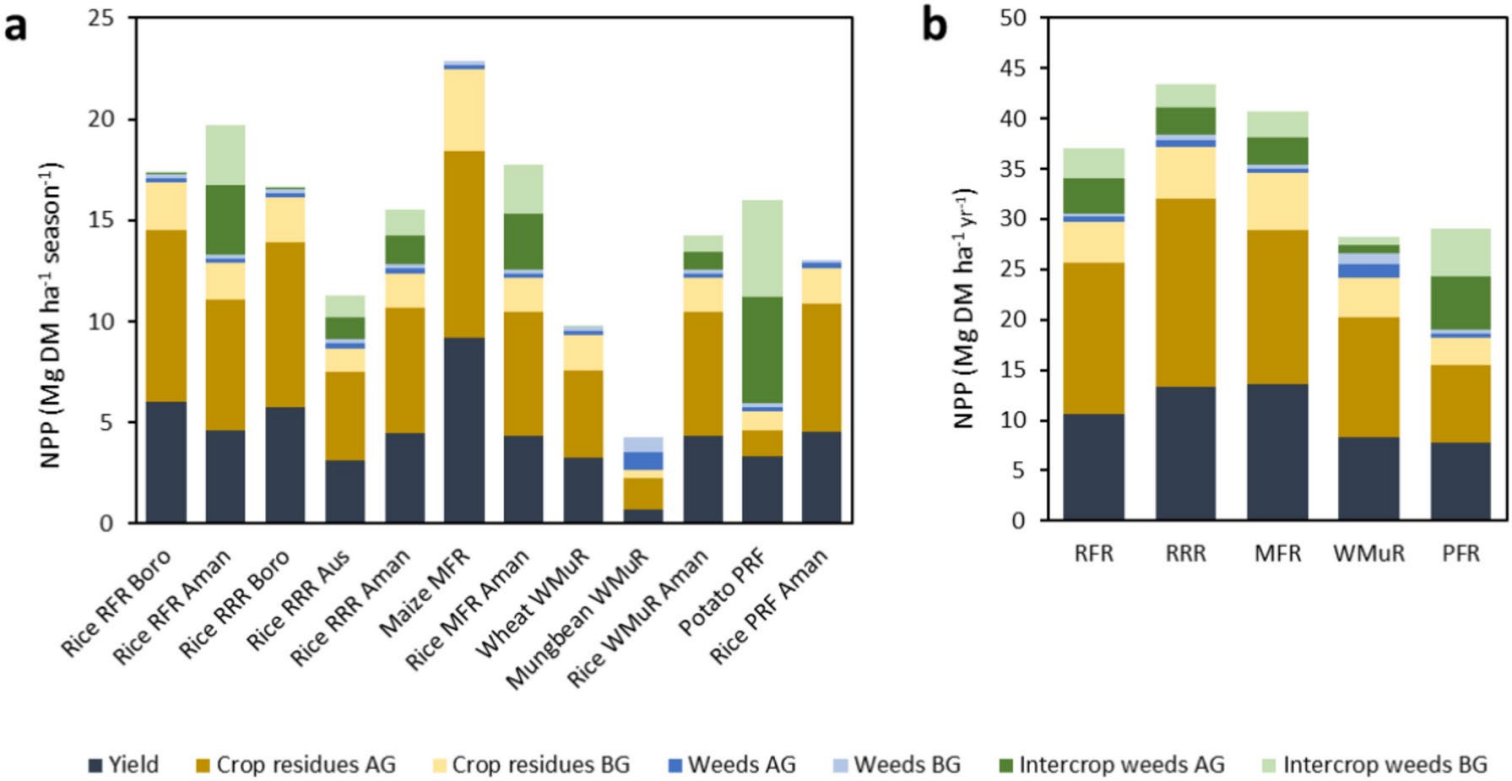
Net primary productivity in the studied conventionally managed crops over their cropping season and intercrop period (**a**) and in their corresponding cropping systems over 1 year (**b**); *DM* Dry Matter, *AG* Above Ground, *BG* Below Ground, *RFR* Rice-Fallow-Rice, *RRR* Rice-Rice-Rice, *MFR* Maize-Fallow-Rice, *WMR* Wheat-Mungbean-Rice, *PFR* Potato-Fallow-Rice.

The R-F-R cropping pattern gave higher dry matter (DM) yield (6.05 Mg DM ha^−1^ season^−1^) in the boro season than in the aman (4.63 Mg DM ha^−1^ season^−1^) season, however the NPP was higher in aman season including the fallow period (Fig. 1a). The NPP was the highest in M-F-R cropping pattern with 22.88 Mg DM ha^−1^ in maize and 17.75 Mg DM ha^−1^ season^−1^ in rice. Weeds were also considered in NPP of crops. Intercrop weed biomass production was present in the crops which had a fallow period before their season. Therefore, in potato-based pattern potato had higher NPP than rice because of having a fallow period before the season. The total NPP was highest in R-R-R (43.42 Mg DM ha^−1^ year^−1^) followed by M-F-R (40.63 Mg DM ha^−1^ year^−1^) and R-F-R (37.05 Mg DM ha^−1^ year^−1^) (Fig. 1b). The W-Mu-R has the lowest average productivity (8.38 Mg DM ha^−1^ year^−1^) with the least yield in mungbean (4.26 Mg DM ha^−1^ year^−1^).

### Nitrogen inputs for crops under different cropping patterns

**Fig. 2:**
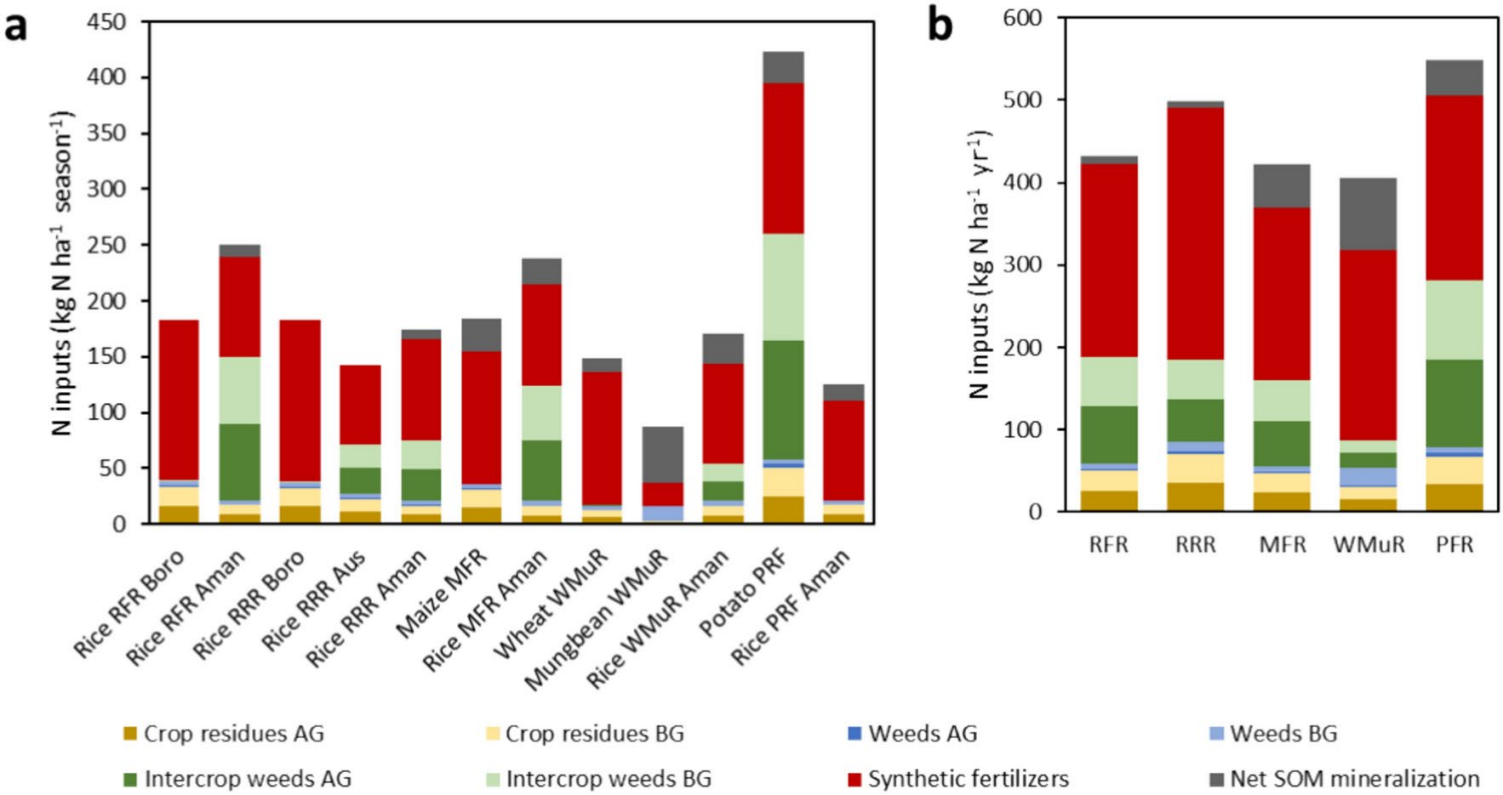
Nitrogen (N) inputs in the studied conventionally managed crops over their cropping season and intercrop period (**a**) and in their corresponding cropping systems over 1 year (**b**); *AG* Above ground, *BG* Below ground, *SOM* Soil organic matter, *RFR* Rice-Fallow-Rice, *RRR* Rice-Rice-Rice, *MFR* Maize-Fallow-Rice, *WMR* Wheat-Mungbean-Rice, *PFR* Potato-Fallow-Rice.

Nitrogen inputs considered for the estimation of direct N_2_O emissions according to IPCC guidelines are shown in Fig. 2. Synthetic fertilizer acted as the largest source of N supply for all the cropping patterns. The highest amount of N input was at maize, with about 17% of synthetic fertilizer used in all crops (Fig. 2a). For both R-R-R and R-F-R cropping patterns the synthetic fertilizer use was higher in boro than other crops, but the total N input was higher in the aman season for R-F-R pattern while boro had higher N input in the R-R-R pattern. However, the R-R-R pattern had larger amount of N input than the R-F-R pattern. The P-R-F pattern had higher (4–21%) N input (549 kg N ha^−1^ year^−1^) than other patterns where M-F-R came as the second largest input of N (528 kg N ha^−1^ year^−1^) (Fig. 2b). In contrast, the least input of N occurred in the W-Mu-R pattern (406 kg N ha^−1^ year^−1^). Both AG and BG crop residue contributed more in R-R-R (71 kg N ha^−1^ year^−1^) than other patterns, P-R-F had the second highest (67 kg N ha^−1^ year^−1^) N input from that source. Synthetic fertilizer supplied around 23–78% of the N supply, while cover crop (AG + BG) contributed approximately 8–36% of the N supply. Highest contribution from weeds in the intercrop period was found from P-R-F (106.13 kg N ha^−1^ season^−1^) and then in R-F-R (69.01 kg N ha^−1^ season^−1^) pattern. In Bangladesh the common cropping pattern is R-F-R but the N input was higher in R-R-R than in R-F-R. The net SOM mineralization had a minor role in most cropping patterns where mungbean had the higher input both as absolute value (59 kg N ha^−1^ year^−1^) and as share of total N input (58%), followed by potato (29 kg N ha^−1^ year^−1^). Between R-R-R and R-F-R pattern the aman season had the SOM mineralized N input (9–10 kg N ha^−1^ year^−1^) which was about 3% and 2% of total input for the cropping patterns, respectively.

### Soil organic carbon balance

**Fig. 3:**
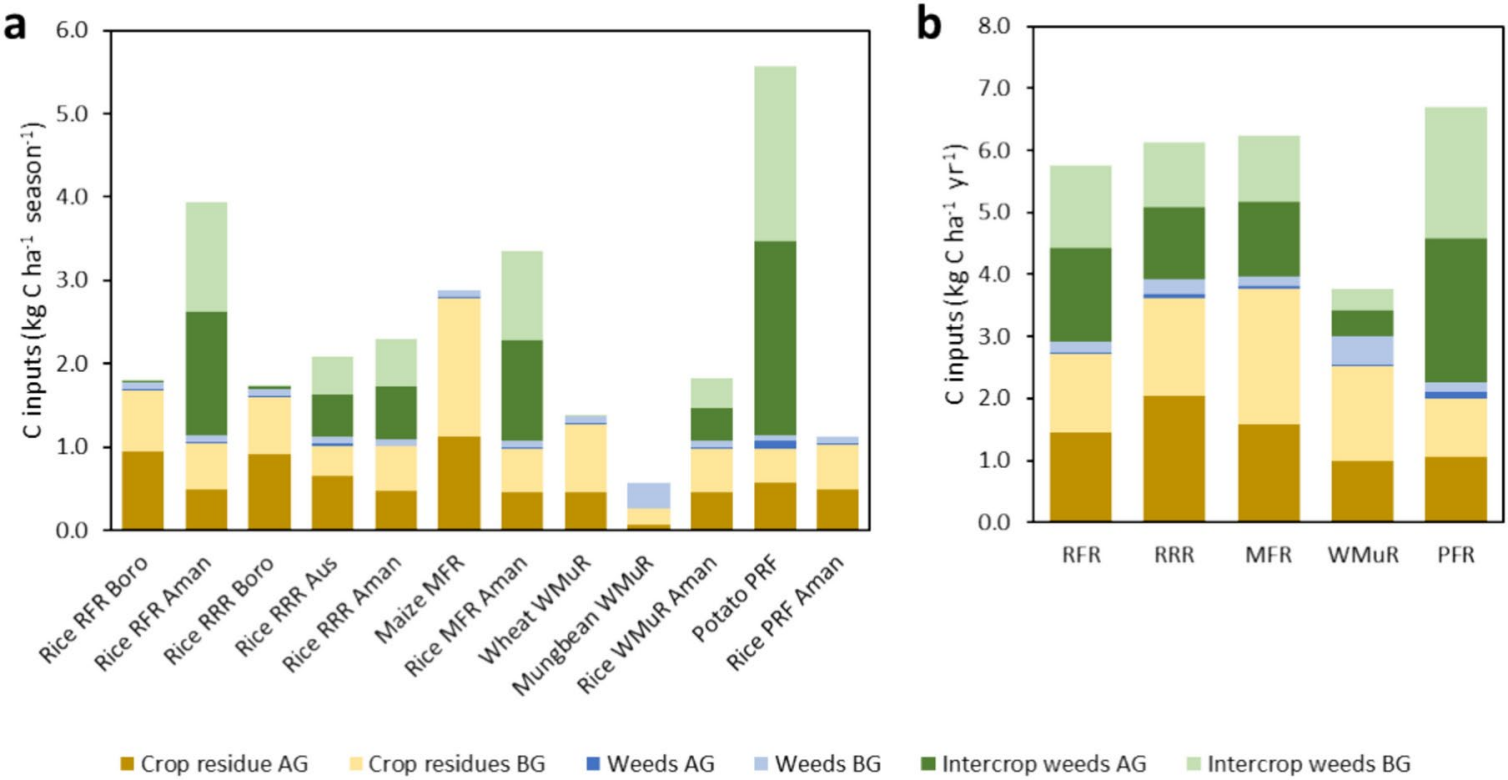
Carbon inputs in the studied conventionally managed crops over their cropping season and intercrop period (**a**) and in their corresponding cropping systems over 1 year (**b**); *AG* Above ground, *BG* Below ground, *SOM* Soil organic matter, *RFR* Rice-Fallow-Rice, *RRR* Rice-Rice-Rice, *MFR* Maize-Fallow-Rice, *WMR* Wheat-Mungbean-Rice, *PFR* Potato-Fallow-Rice.

As the crops were conventionally managed, organic fertilization was not practiced. Weeds, crop residues and cover crops (AG and BG) contributed to the C input in all the cropping patterns. The P-R-F had the highest C in put (7 kg C ha^−1^year^−1^) while W-Mu-R had the lowest (4 kg C ha^−1^ year^−1^). C input was about 7% higher in R-R-R (6.13 kg C ha^−1^ year^−1^) than R-F-R (5.76 kg C ha^−1^ year^−1^). Carbon input in boro rice for both R-R-R and R-F-R was about 91–92% through crop residues and rest amount came from weeds and cover crops. Weeds and cover crops grew during the fallow period (Fig. 3) where the crop residue was the largest source of C input for the cropping patterns. The P-R-F, M-F-R and R-R-R patterns had about 5–14% higher C input than R-F-R pattern. Humified C input was higher in P-R-F (Fig. 3) than other patterns and it was about 14% higher than in R-F-R.

**Fig. 4:**
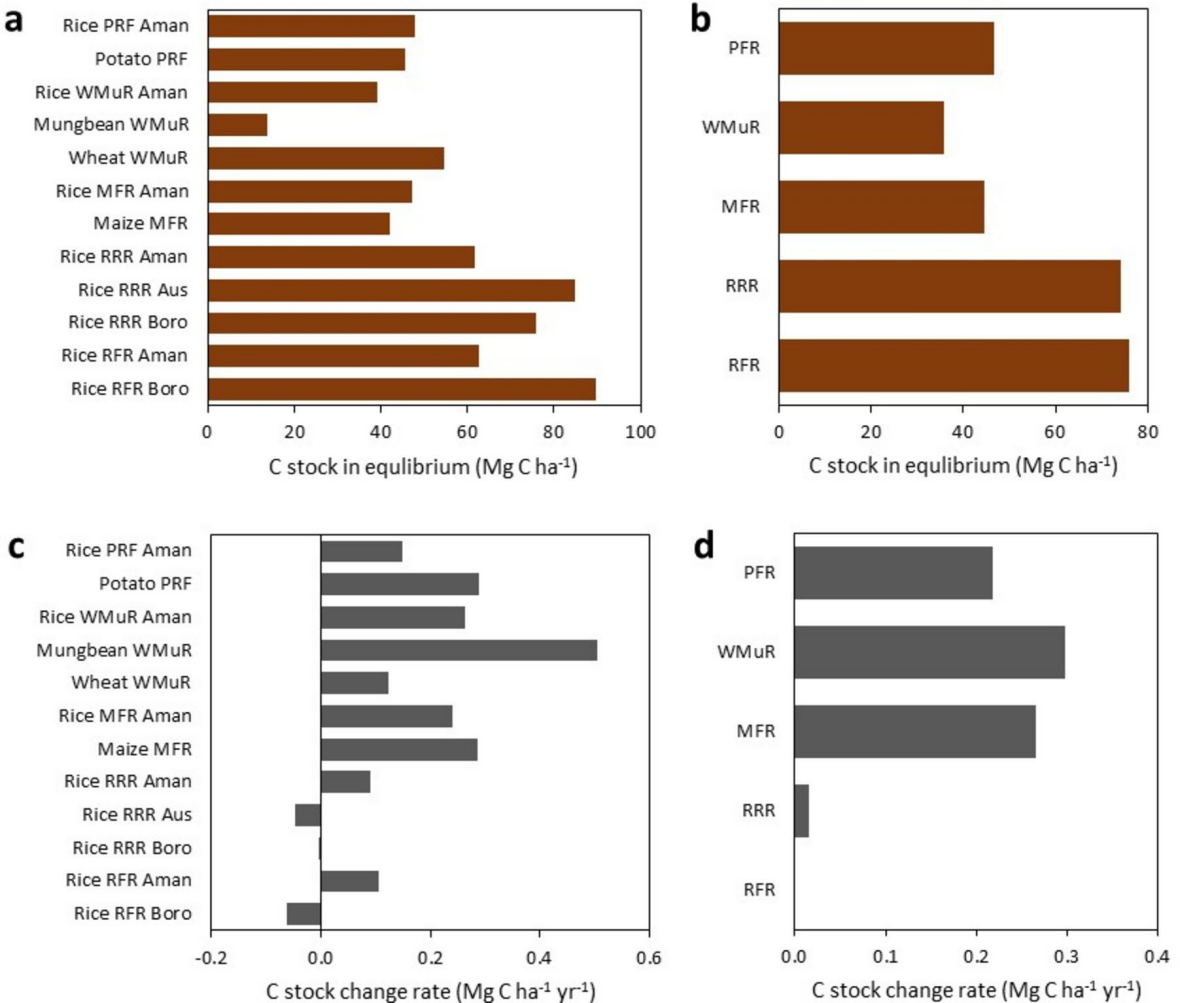
Carbon stock in equilibrium in the studied crops (**a**) and cropping systems (**b**), and C stock change rates in the studied crops (**c**) and cropping systems (**d**). *RFR* Rice-Fallow-Rice, *RRR* Rice-Rice-Rice, *MFR* Maize-Fallow-Rice, *WMR* Wheat-Mungbean-Rice, *PFR* Potato-Fallow-Rice.

The C stock in equilibrium ranged from 13–90 Mg C ha^−1^ (Fig. 4a,b). The boro from R-F-R season had the highest stock (90 Mg C ha^−1^) (Fig. 4a). The highest C stocks among cropping patterns was achieved in the reference rotation, the R-F-R (76.1 Mg C ha^−1^), similar to the R-R-R (74.3 Mg C ha^−1^) (Fig. 4b). The R-F-R pattern had 41%, 29% and 38% higher C stock than M-F-R, W-Mu-R and P-R-F patterns, respectively. Aman rice stock had more C than potato and maize but less than wheat in R-F-R. The values of C stock change rate in the studied crops ranged from − 0.06 in the boro rice from the reference cropping pattern R-F-R to 0.51 Mg C ha^−1^ year^−1^ in mungbean (Fig. 4c). The R-R-R cropping pattern had zero C stock rate change, reflecting our choice of this pattern as the reference one (Fig. 4d), while the R-R-R pattern also had a value close to zero (0.02 Mg C ha^−1^ year^−1^). All other rotations involved the cultivation of non-flooded crops and were associated to lower C stocks (Fig. 4b) and subsequently to net C loss, all presenting similar C stock change rates ranging 0.22–0.3 Mg C ha^−1^ year^−1^.

### Soil fluxes of trace greenhouse gases

**Fig. 5:**
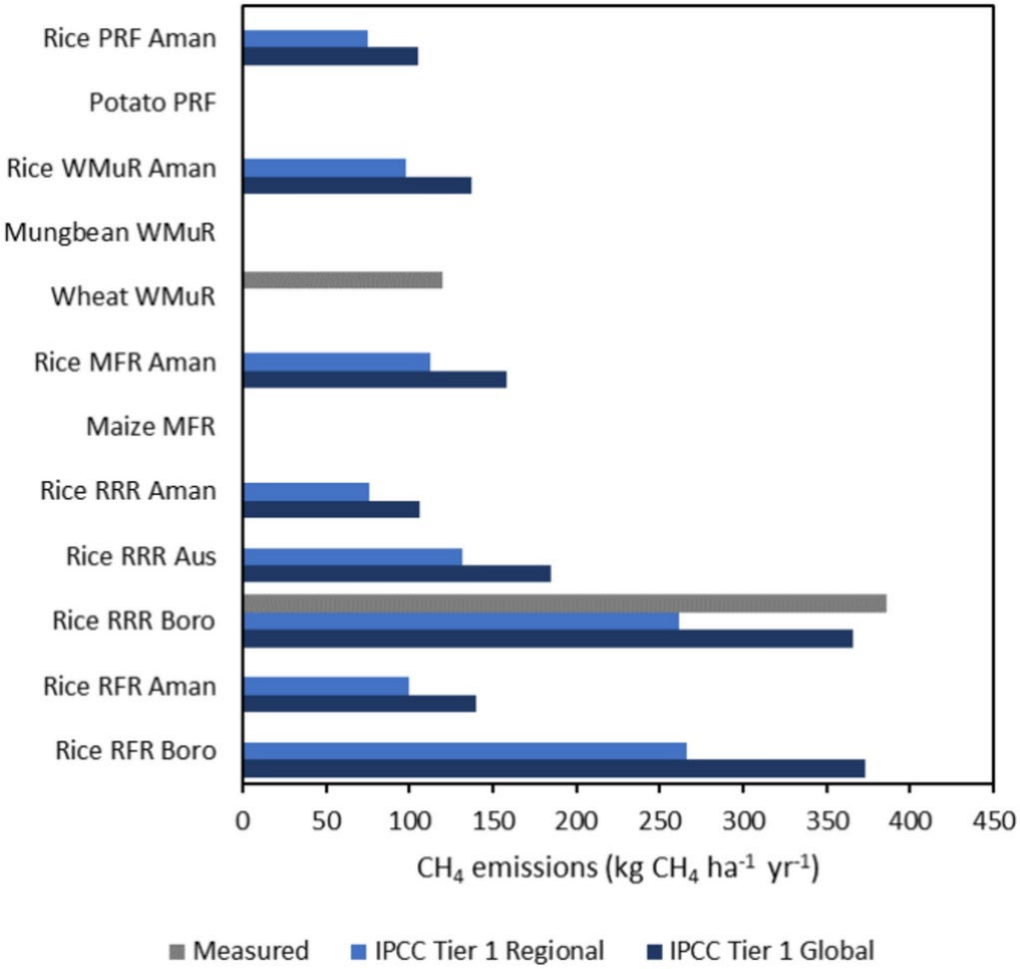
Comparison of three different approaches to estimated methane (CH_4_) emissions from the studied crop fields, including field measured emissions, IPCC Tier 1 approach using the Global baseline emission factor, and IPCC Tier 1 approach using the Regional-specific baseline emission factor for South Asia. *RFR* Rice-Fallow-Rice, *RRR* Rice-Rice-Rice, *MFR* Maize-Fallow-Rice, *WMR* Wheat-Mungbean-Rice, *PFR* Potato-Fallow-Rice.

The IPCC guidelines for the calculation of soil CH_4_ emissions take only rice cultivation into account, as it is assumed to be the only crop grown under waterlogging conditions. However, in field level we measured CH_4_ emissions from boro rice and from wheat fields (Fig. 5). The comparison of the boro rice measured emissions with the IPCC-based estimates shows that the IPCC Tier 1 approach using the Global baseline emission factor resulted in a very similar value (366 kg CH_4_ ha^−1^ year^−1^) to measurements (385 kg CH_4_ ha^−1^ year^−1^), while the value obtained using the region-specific baseline emission factor for South Asia was much lower (262 kg CH_4_ ha^−1^ year^−1^). Therefore, we decided to use the Global baseline emission factor in the calculation of C footprints. The wheat field measured emissions were 120 kg CH_4_ ha^−1^ year^−1^, which is even more than our estimated Global baseline emissions for aman rice in R-R-R (106 kg CH_4_ ha^−1^ year^−1^). The IPCC Tier 1 Global values estimated across all crops indicated that among the rice seasons boro caused the highest emissions (366 and 373 kg CH_4_ ha^−1^ year^−1^ in R-R-R and R-F-R, respectively) followed by aus (185 kg CH_4_ ha^−1^ year^−1^ in R-R-R) and then aman (106–158 kg CH_4_ ha^−1^ year^−1^ across all rotations).

**Fig. 6:**
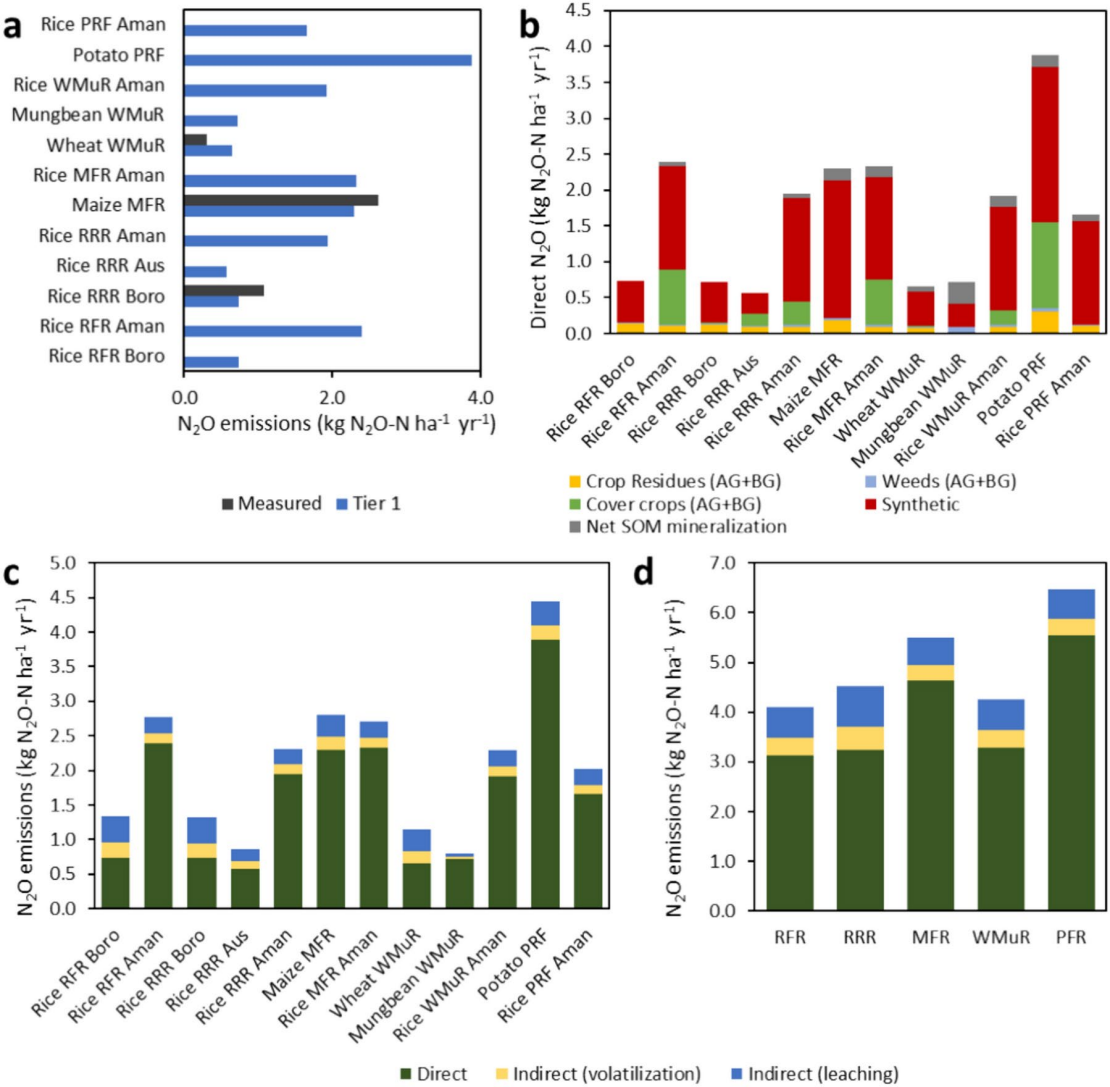
Nitrous oxide (N_2_O) emissions in the studied crops, including direct N_2_O emissions comparing measured and IPCC Tier 1 estimations (**a**), direct N_2_O emission sources by crop (**b**), total N_2_O emission by emission type and crop (**c**) and total N_2_O emissions by emission type and cropping system (**d**). *RFR* Rice-Fallow-Rice, *RRR* Rice-Rice-Rice, *MFR* Maize-Fallow-Rice, *WMR* Wheat-Mungbean-Rice, *PFR* Potato-Fallow-Rice.

Measured data for N_2_O loss was only available for boro, maize and wheat field, and these values compared well with the ones calculated using the IPCC (2019) Tier 1 default emission factors (Fig. 6a). The Tier 1 estimate for boro rice is 0.73 kg N_2_O–N ha^−1^ year^−1^ while the measured value was 1.08 kg N_2_O–N ha^−1^ year^−1^. The Tier 1 value of N_2_O emissions for maize was 2.3 kg N_2_O–N ha^−1^ year^−1^ but the measured value was 2.62 kg N_2_O–N ha^−1^ year^−1^. In wheat field 0.65 kg N_2_O–N ha^−1^ year^−1^ emitted as N_2_O but the measured value was 0.31 kg N_2_O–N ha^−1^ year^−1^. Thus, the measured N_2_O emissions values were 1.47 and 1.13 times higher than the Tier 1-estimate under rice and maize, respectively, while in wheat the measured data was 2.09 times lower than the Tier 1 value.

Synthetic fertilizers acted as the largest source of N_2_O emission, about 44–90% of total emissions in the studied (Fig. 6b). The highest emissions were in M-F-R (6.31 kg N_2_O–N ha^−1^ year^−1^) and P-R-F had the second highest (5.54 kg N_2_O–N ha^−1^ year^−1^). In R-F-R, synthetic fertilizers had the least (3.13 kg N_2_O–N ha^−1^ year^−1^) amount of N_2_O emission occur where aman caused about 76% emissions. In boro and aman seasons crop residue caused about 18% and 4% of emissions, respectively. It could be due to 1–2% of emissions from weeds and crop residues in this pattern. The second lowest emissions were from R-R-R. However, M-F-R emitted 1.15–2.6 times higher N_2_O emissions than other crops. The potato-based pattern contributed to the second highest amount of total direct N_2_O emission (5.54 kg N_2_O–N ha^−1^ year^−1^) where 23% of emissions occur from synthetic sources. (Fig. 4a). The W-Mu-R pattern was the third largest source of total direct N_2_O emission with 3.31 kg N_2_O–N ha^−1^ year^−1^. The dry land crops maize, potato had higher emissions than wet land crops.

Among three different types of emission, direct N_2_O emission was the dominant pathway across cropping patterns, with emissions being 3–19 times and 2–10 times higher than volatilization and leaching, respectively (Fig. 6c,d). Between volatilization and leaching loss, higher N_2_O emissions through volatilization were estimated for all the crops. In case of mungbean (1.25 kg N_2_O–N ha^−1^ year^−1^) (Fig. 6c) small leaching losses caused the lowest total emission in W-Mu-R (6.18 kg N_2_O–N ha^−1^ year^−1^) whereas R-R-R had the largest emissions, approximately 14 kg N_2_O–N ha^−1^ year^−1^ (Fig. 6d). The M-F-R pattern holds the second position in terms of overall emission value, with higher emissions in maize (5.83 kg N_2_O–N ha^−1^ year^−1^).

### Global warming potential (GWP) and C footprint

**Fig. 7:**
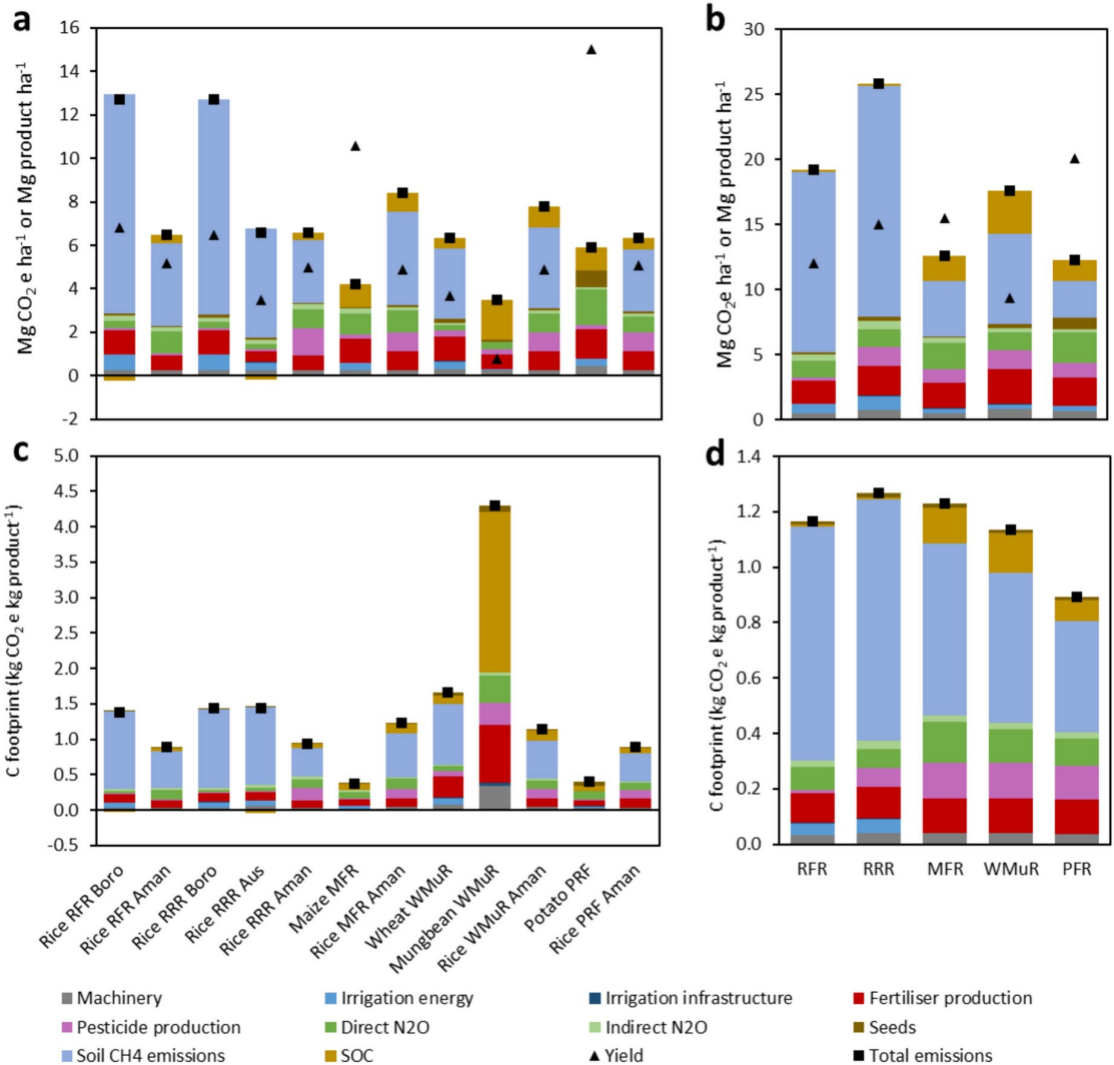
Area-based greenhouse gas (GHG) emissions (kg CO_2_e kg ha^−1^) and yield (Mg ha^−1^) of studied products in all crop seasons (**a**) and over the full year in each cropping pattern (**b**), Carbon (C) footprint (kg CO_2_e kg product^−1^) of studied products in all crop seasons (**c**) and weighted average C footprint of rice in each crop pattern (**d**). *RFR* Rice-Fallow-Rice, *RRR* Rice-Rice-Rice, *MFR* Maize-Fallow-Rice, *WMR* Wheat-Mungbean-Rice, *PFR* Potato-Fallow-Rice.

Major portion of the area-based GWP was due to soil CH_4_ emission from rice and wheat-based cropping patterns, which was about 50–80% of total GWP (Fig. 7a). The GWP varied with the studied crops for different cropping patterns. All the predictors impacted on the GWP. The R-F-R pattern decreased the GWP about 25% over the R-R-R pattern. Among the crops boro in R-F-R (12.72 Mg ha^−1^) and R-R-R (12.69 Mg ha^−1^) had higher GWP than other crops (Fig. 7a). The GWP followed the trend boro < aman < aus < wheat < maize < potato < mungbean.

Boro rice in both R-F-R and R-R-R pattern generated the highest CH_4_ emissions (ca. 10.1–9.9 Mg CO_2_e ha^−1^) accounting for 77–79% of total emissions, and resulting in the highest GWP among the studied crops. Therefore, the R-R-R and R-F-R cropping patterns, the only ones including boro rice, had the highest total GWP, with 25.8 and 19.2 Mg CO_2_e ha^−1^, respectively (Fig. 7b). Maize, potato and mungbean had no soil CH_4_ emissions. Though wheat is a dry land crop, the cropping pattern containing wheat (W-Mu-R) had higher GWP than other two cropping patterns containing dry land crops, such as potato and maize, due to CH_4_ emissions resulting from water-logging conditions. The total GWP by crop rotation varied from 12.3 Mg ha^−1^ in P-R-F to 25.8 Mg ha^−1^ in R-R-R (Fig. 7b). The P-F-R and M-F-R had the lowest GWP (12.3 and 12.6 Mg CO_2_e ha^−1^). Fertilizer production was the second-largest source of total emissions with 8–30%. The highest N_2_O emissions were found in the P-R-F pattern (2.6 Mg CO_2_e ha^−1^) and then in M-F-R (2.1 Mg CO_2_e ha^−1^) pattern and the lowest was in W-Mu-R (1.7 Mg CO_2_e ha^−1^) pattern. Machinery and seed impeded effect on the GHG balance mostly in P-R-F pattern (Fig. 7b) while it was similar for all the rice seasons (Fig. 7a) in every pattern. The contribution of pesticide production was higher in R-R-R (1.42 Mg CO_2_e ha^−1^) and W-Mu-R (1.40 Mg CO_2_e ha^−1^) and lower in R-F-R (0.2 Mg CO_2_e ha^−1^). Pesticide production contributed largely in aman season from R-R-R had higher C emissions. During rice season the irrigation energy contributed to C emissions. Considering all cropping patterns, the role of SOC was relatively minor in the GWP, which was the result of relatively small differences in the SOC balance as compared to the reference cropping pattern R-F-R. The highest share was observed for mungbean in which SOC related CO_2_ emissions represented 50% of the GWP due to the combination of high C mineralization, low C input, and low levels of the other emissions. Net C sequestration was found in boro (R-F-R) and aus (R-R-R) only, although it only compensated 1% of the GWP (Fig. 7b).

Large differences were found in the C footprints of the studied crops, with one order of magnitude difference between the extreme values (0.4–4.3 kg CO_2_e kg product^−1^, Fig. 7c). These differences can be explained by the different levels of GHG emissions per unit area but also by yields. In the case of rice production systems, the lowest C footprint (0.9 kg CO_2_e kg product^−1^) was observed in the aman R-F-R, while the highest was observed in the boro (1.44 kg CO_2_e kg product^−1^) and aus (1.44 kg CO_2_e kg product^−1^) of the R-R-R rotation (Fig. 7c). Therefore, the high yield of the boro rice could not compensate the high area-based emissions of this system. Cropping patterns containing dryland crops obtained higher yields than other rice-based patterns (Fig. 7d). Potato and maize-based patterns had the largest production, roughly 20.1 Mg product ha^−1^ year^−1^ and 15.48 Mg product ha^−1^ year^−1^, respectively. These high yields, in combination with relatively low area-based emissions due to the absence of soil CH_4_ emissions, led to maize and potato having the lowest C footprint of all studied crops (0.4 kg CO_2_e kg product^−1^). Conversely, the lowest yield was observed in mungbean (0.8 Mg product ha^-1^) and mungbean-based pattern (9.4 Mg product ha^−1^ year^−1^), which resulted in the highest C footprint (4.3 Mg CO_2_e kg product^−1^).

## Discussion

This study presents a comprehensive assessment of the C footprint of major cropping systems in Bangladesh through an attributional LCA approach, utilizing an Excel-based C footprint calculation tool. The findings highlight significant variations in GHG emissions, C sequestration, and overall C footprint across different cropping patterns. The analyses revealed that cropping patterns incorporating rice, particularly the R-R-R and R-F-R systems, exhibited the highest total GWP due to substantial CH₄ emissions. Emissions driven primarily by CH_4_ emissions from boro rice which accounted for 77–79% of total emissions. In support to our result, Rahman et al.^29^ reported that during rice cultivation CH_4_ emissions contributed to about 77% of total emissions. Comparatively, the R-F-R pattern had a lower GWP, reflecting a 25% reduction over the R-R-R pattern, likely due to lower CH₄ emissions in the aman season and a smaller overall N input. Carbon input was the highest for the R-F-R and R-R-R patterns where the inputs were mostly done through crop residues, and weeds and according to this, the R-R-R and R-F-R patterns showed the highest soil CH_4_ emissions. These emissions were also present in all rice treatments and in wheat. Our finding of significant soil CH_4_ emissions in wheat cultivation (representing 51% of the GWP of this crop) is, to our knowledge, unprecedented in the literature, and indicates the need to study the extent of wheat cultivation under these soil conditions in Bangladesh and in other parts of the world, to know the magnitude of these soil CH_4_ emissions. The emissions were absent in maize, potato and mungbean crop. In conventional practice rice is grown under continuous flooding (anaerobic) conditions which is the prerequisite for CH_4_ emissions. Methane is the final product of anaerobic breakdown of SOM by the action of methanogens in wetland paddy field since absence of oxygen is required for the function of methanogens. On flooding, short-term evolution of hydrogen immediately follows the disappearance of oxygen, CO_2_ increases, and, with decreasing CO_2_, CH_4_ formation increases^30^. In R-R-R and R-F-R cropping pattern the cover crops, crop residues and weeds were present where they act as C source. In this study the SOC balance exhibited variations across cropping patterns, with the R-F-R and R-R-R rotations maintaining higher SOC stocks compared to the other systems. However, the overall contribution of SOC changes to the GWP was relatively minor, accounting for only 1% of the total emissions in boro and aus crops. The rate and pattern of organic matter addition and decomposition determine the rate and pattern of CH_4_ production. Large portions of CH_4_ formed in an anaerobic soil due to anaerobic degradation of SOM, CH_4_ may remain trapped in the flooded soil. Entrapped CH_4_ may be oxidized to CO_2_ when the floodwater is drained during the rice growing season or when the soil dries at the end of or after the rice growing season. But large amounts of entrapped CH_4_ may escape to the atmosphere immediately after the floodwater recede. Wheat also needs less irrigation, but the studied field was moist throughout the season due to high water table, which stimulated CH_4_ emissions. Therefore, crop residues through soil CH_4_ emissions have contributed to the highest CO_2_e emissions. Similar results were found by Vu et al.^31^ in their study, the lowest CH_4_ emissions were found in mineral fertilizer compared to the highest value for farmyard manure and compost manure. This is due to the inclusion of materials that are rich in quickly biodegradable organic matter offering readily biodegradable C for CH_4_ synthesis^31^. Yagi and Minami^32^ found that the average value for CH_4_ flux in rice straw was higher in comparison to the compost and mineral-treated plots. Zhang et al.^33^ found that residue retention increased CH_4_ emission by two times compared to the paddy fields where residue retention did not take place. Sanchis et al.^34^ reported that continuously flooded rice fields without any added straw produced average CH_4_ emissions that were 93% higher compared to rainfed, intermittently flooded, or non-flooded irrigated water management. That means, independent of the addition of organic matter to the soil, continues flooding can foster suitable conditions for CH_4_ production. However, organic fertilizers and flood irrigation also promote C sequestration, which can result in reduced net GHG emissions despite higher CH_4_ emissions^35^. In the R-F-R pattern, there was a fallow period, and also the SOC sequestration for boro rice was approximately 0.23 Mg CO_2_ ha^−1^ and aus sequestrated 0.17 Mg CO_2_ ha^−1^ in R-R-R though the emissions were the highest for rice crop. As a result, R-F-R and R-R-R had higher CH_4_ emissions but they were also the only crops which were able to sequester C in soil. In our study, the estimated magnitude of this sequestration was very low in terms of GWP compared to CH_4_ emissions, but long-term field studies are needed to verify these results and to assess SOC changes in the R-F-R rotation, which we assumed to be at equilibrium.

Methane is the main component of the GHG balance and C footprints of the studied crops cultivated under flooded or waterlogged conditions, including rice but also wheat. The dominance of soil CH_4_ emissions in the C footprint of rice is well established in the literature. For example, Poore and Nemecek^36^ found that soil CH_4_ emissions represented 28–82% of life cycle rice GHG emissions in a comprehensive global meta-analysis. This result is also in line with previous LCA studies. For example, Alam et al.^21^ found that on-farm CH_4_ emissions were the largest contributor to overall GHG emissions in the monsoon paddy rice. Another LCA estimation in southeast India showed that reduced water use in the System of Rice Intensification reduced GHG emissions^37^. They also verified that, regardless of retained residue levels, CH_4_ is produced during the organic matter decomposition process in anaerobic soil conditions in the profile of both puddled and non-puddled submerged fields. In non-flooded crops the GHG was mostly attributed to CF application. The C footprint values of paddy rice production found in our study, ranging 0.9–1.46 kg CO_2_e kg^−1^, are lower than the global median values (2.4 and 1.68 kg CO_2_e kg^−1^, after converting the value from milled rice) reported by Poore and Nemecek^36^ and Clune^38^ respectively. Our results are also lower to those of another study in Bangladesh (3.15 kg CO_2_e kg^−1^) but similar to other studies in this country, e.g., 1.11–1.57 kg CO_2_e kg^−1^ by Alam et al.^21^, and 1.35 kg CO_2_e kg^−1^ (after converting from milled rice). In Bangladesh about 293 kg CO_2_e ha^−1^ day^−1^ GWP was found in boro rice growing season^6^ and in this study it was about 13 Mg CO_2_e ha^−1^ emissions. We found 4.20 Mg CO_2_e ha^−1^ emissions in maize where few references are provided here where they found about 4.9 Mg CO_2_e ha^−1^ emissions^39^ in Bangladesh, 3.4 Mg CO_2_e ha^−1^ emissions in India and 14.8 Mg CO_2_e ha^−1^ emissions^33^ in China.

On the other hand, synthetic fertilizers emerged as the primary source of N inputs across all cropping systems, contributing between 23 and 78% of total N supply. The increased use of synthetic fertilizers in maize-based systems (M-F-R) and potato-based systems (P-R-F) correlated with higher N₂O, another climate relevant GHG, reinforcing the well-established link between fertilizer application and N₂O production. Direct N_2_O emissions were about half in rice and wheat (1.44 kg N_2_O–N ha^−1^ year^−1^ on average), which were cultivated under water-logging conditions, then in dry land crops also intensively fertilized such as maize and potato (3.09 kg N_2_O–N ha^−1^ year^−1^ on average). Maize emits 1.66 to 4.09 MT CO_2_eq GHGs in growing seasons^39^. Emissions of N_2_O are the result of microbial nitrification and denitrification in soils, controlled principally by soil water and mineral N contents, labile organic C, and temperature^40^. Transformational modifications (anaerobic rice systems into aerobic) in rice cultivation practices sustain yield but at the cost of higher N loss with high N_2_O emissions. In dry land the aerobic condition facilities the nitrification process (ammonium-nitrite-nitrate), after irrigation (anaerobic condition) which provides the substrates of denitrification (nitrate-nitrogen dioxide-nitrous oxide) in crops with transitional (aerobic-anaerobic) water state condition. Cropping patterns with dryland crops, such as maize and potato, exhibited lower GWP and carbon footprints due to negligible CH_4_ emissions, with maize-based systems also showing the second-highest N inputs. However, mungbean, despite its low yields, had the highest carbon footprint, mainly due to a combination of higher N_2_O emissions and lower productivity. For a rice-based cropping system, Islam et al.^14^ reported the effect of fertilizer, 50% from urea (synthetic) and 50% from poultry litter, on GHG emissions from rice fields during the aus and aman seasons in Bangladesh. According to Jahangir et al.^6^, cumulative N_2_O emissions during the growing season increased significantly with increasing N application rates. Mazz et al.^41^ reported from their meta-analysis that intensive rice system and SOC increase N_2_O emissions consistently, however, rice system has 57% lower emissions than other cereals while, maize has 71% higher N_2_O emissions than rice in Asia-Africa and the emissions increase by 5% with each percent increase in SOC. Chemical source of N also increases N_2_O emissions and about 0.4% increase of N_2_O is associated with addition of 1 kg N ha^−1^. Results from an increasing number of experiments using different N fertilizer rates showed that emissions of N_2_O respond exponentially to increasing N inputs in a variety of soil types, climates, and fertilizer formulas^42^.

However, the IPCC tier 1 method that we have applied in this work is based on fixed emission factors of the applied N (depending on climate, flooding conditions and input type), which implies a linear relationship between N inputs and emissions. Therefore, more field studies are needed in Bangladesh and similar areas to improve N_2_O estimations in inventories and in LCA studies. Compared to all the four patterns R-F-R had the lowest N_2_O emissions (3.13 kg N_2_O–N ha^−1^ year^−1^) may be attributed to the fallow period where the fallow period had only spontaneous weed growth with lower emissions due to lack of substrate for denitrification but cropping pattern with three crops had emissions from each crop. However, contradictory statement from numerous studies stated that the N_2_O emissions from fertilized paddy fields during the fallow season is significantly larger than the N_2_O emissions during the cropping season^43^. According to a global meta-analysis by Chen et al.^44^, crop residues generated equivalent to or more N_2_O emissions than synthetic fertilizers, but another meta-analysis showed much lower emissions from crop residues. However, according to Shan and Yan^45^, the addition of crop residue with synthetic fertilizer reduced N_2_O emissions by 11.7% when compared to synthetic fertilizers alone. There is a lack of data on indirect N_2_O emissions from different crop fields. Loss of N through volatilization and leaching are not included in many studies but they can represent an important contribution to fertilizer-related global warming through indirect N_2_O emissions^46^.

These values were used as total emissions for a crop since there was no regional data available in Bangladesh, there were huge variations in GHG emissions because of field management, seasonal variation, residue management and mostly fertilization rates. The participative approach to develop the C footprint calculation tool allows for the incorporation of a wide range of perspectives and expertise, resulting in a comprehensive and accurate assessment of GHG emissions. It is also highly versatile, with the possibility to incorporate changes in the assumptions, parameters, and data that are used in the calculations. This tool can be useful for identifying opportunities for reducing GHG emissions. By providing a detailed understanding of the sources of GHG emissions, as well as C and N flows, a C footprint calculation tool can help identify specific areas where changes in practices or technologies can lead to significant reductions of such emissions. This can be especially valuable for policymakers and industry representatives, as it can inform the development of more effective and targeted climate impact policies and strategies for reducing emissions. However, this tool is not a one-time solution but a continuous process, it needs to be regularly updated to reflect the latest research and data, and new technologies and practices that may emerge.

## Conclusions

We developed and used a carbon footprint calculation tool to estimate the GHG emissions and C balance from typical cropping systems in Bangladesh. Rice-based cropping pattern with dryland crops had higher N_2_O than sole rice-based cropping systems but CH_4_ emissions were higher in sole rice-based patterns, resulting in higher GHG emissions and C overall footprint. Methane contributed about 50–80% of total GHG emissions from upstream–downstream and crop production. Among the rice-based cropping systems, boro and aus from R-F-R and R-R-R patterns sequestrated C in soil, although this had a negligible effect on the C footprint. A novel finding of this study is the presence of CH_4_ emissions from wheat field, as the field was under moist condition throughout the season. The IPCC default Tier 1 value was only available for rice seasons (aus, aman and boro) and measured data was only available for boro and wheat so further studies are required for validation and developing suitable GHG mitigation strategies in agricultural fields in Bangladesh.

## Supplementary Information

Supplementary Information.

## Data Availability

The datasets used and/or analysed during the current study are available at https://dataverse.harvard.edu/api/access/datafile/10546398.
