# Supplementary material for: Carbon footprint and greenhouse gas emissions of different rice-based cropping systems using LCA
Source: Sci Rep. 2025 Mar 25;15:10214. doi: 10.1038/s41598-025-90157-2 (PMC11937248; doi:10.1038/s41598-025-90157-2)
Supplement: Supplementary file 1 — Supplementary Information. [file 41598_2025_90157_MOESM1_ESM.docx]

# Supplementary information

## Allocation of the Product Value

Based on product value, products shared 79–100% of the greenhouse gas emissions in different crops, while residues shared the rest of the percentage (Suppl. 1). As residue of maize, mungbean, and potato have no price/market value, it has no contribution in the systems, whereas the products contribute 100% due to its product value. Among rice, rainfed T. aman rice residue has the highest contribution as it’s large proportion (90%) is used as co-product and high price of residue in Bangladesh. Supplementary data associated with this article is presented in Suppl. 1.


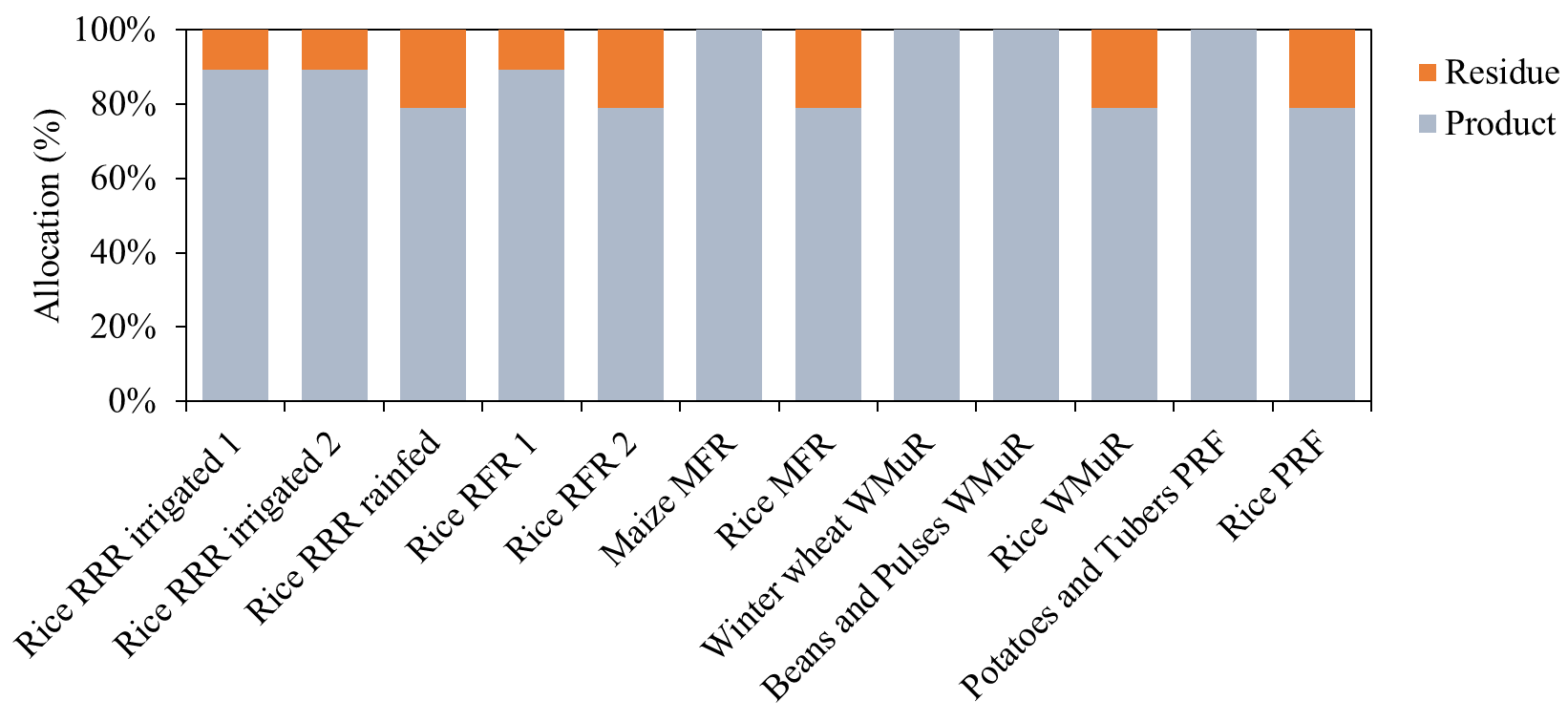


***Suppl. 1*** Allocation of the product value for GHG emissions
